# Quantitative Evaluation of the Invasion Depth of Colorectal Cancer Located on a Colorectal Fold Through the Width of Colorectal-Fold Lateral Contour Using a Lateral Split-View Computed Tomographic Air-Contrast Enema Image

**DOI:** 10.7759/cureus.87941

**Published:** 2025-07-14

**Authors:** Mitsutoshi Miyasaka, Toshio Muraki, Yusuke Nishimuta, Eiji Oki, Kousei Ishigami, Daisuke Tsurumaru

**Affiliations:** 1 Gastrointestinal Endoscopy, Kyushu Cancer Center, Fukuoka, JPN; 2 Clinical Radiology, Kyushu University, Fukuoka, JPN; 3 Surgery and Science, Kyushu University, Fukuoka, JPN

**Keywords:** colorectal cancer, computed tomographic air-contrast enema, computed tomographic colonography, lateral deformity, lateral split-view

## Abstract

Purpose: The aim of the study was to investigate the usefulness of quantitatively evaluating the width of lateral contour on a lateral split-view computed tomographic air-contrast enema (CT enema) image to diagnose the invasion depth of colorectal cancer (CRC) located on a colorectal fold.

Methods: The cases of 22 patients with 22 fold-located CRCs, that is, 12 (54.5%) early CRCs and 10 (45.5%) advanced CRCs, who underwent a pretherapeutic CT colonography, were retrospectively examined. T1-stage CRCs were classified into two categories according to the Japanese guideline: T1a-stage (carcinoma invading the superficial submucosa (<1000 μm)) and T1b-stage (carcinoma invading the deeper submucosa (≧1000 μm)). The maximum width of colorectal-fold lateral contour on which the CRC was located, i.e., the gap distance between the two adjacent haustrations, was calculated from the lateral split-view CT enema image by three gastrointestinal radiologists. These values were compared between the intramucosal/T1a CRCs and the T1b/more deeply invading CRCs. The inter-rater intraclass correlation coefficients were also evaluated for reliability.

Results: The maximum widths of colorectal-fold lateral contour were significantly higher in the T1b/more deeply invading CRCs than in the intramucosal/T1a CRCs (3.3±1.4 mm and 12.1±4.6 mm, respectively; p<0.0001). The optimum cut-off value of the maximum width of colorectal-fold lateral contour for differentiating the former from the latter was 6 mm, with a sensitivity and specificity of 92.9% and 87.5%, respectively. The inter-rater intraclass correlation coefficient for the measurement of colorectal-fold lateral contour was 0.949.

Conclusions: We demonstrated for the first time that the quantitative evaluation of the width of the lateral contour using a lateral split-view CT enema image can improve the diagnostic accuracy of the invasion depth for CRCs located on a colorectal fold.

## Introduction

The invasion depth of colorectal cancer (CRC) lesions is closely related to their risk of metastasis. Surgical resection is the only curative treatment for CRCs with massive submucosal or deeper invasion without distant metastasis, whereas endoscopic resection is the established therapy for intramucosal CRCs or CRCs invading the superficial submucosa (<1000 μm) because they have no risk of metastases [1-4]. In the Japanese Classification of Colorectal Carcinoma 9th Edition, T1-stage CRCs were classified into two categories: T1a-stage (carcinoma invading the superficial submucosa (<1000 μm)) and T1b-stage (carcinoma invading the deeper submucosa (≧1000 μm)) [5]. Therefore, accurate diagnosis of CRC invasion depth, especially differentiation between T1a-stage CRCs and T1b-stage CRCs, is crucial for determining the appropriate therapeutic strategy in CRC in Japan.

Computed tomographic colonography (CTC) is performed worldwide as a preoperative study in CRCs [6-9]. A lateral deformity on CT air-contrast enema (CT enema) or double-contrast barium enema (DCBE) has been shown to be useful in diagnosing the invasion depth of CRCs [10-13]. However, diagnosis of invasion depth based on a lateral deformity may lead to overdiagnosis because the depth of lateral deformity on a CT enema or DCBE image in CRC is understood to depend on both the tumor geometry (mainly with respect to the size of the tumor base) and tumor invasion [14-18]. A CT enema image with cross-sectional multiplanar reconstruction (CS-MPR) images is useful to estimate the depth of lateral deformity based on the tumor geometry in colorectal polyps [19]. Additionally, the quantitative evaluation of lateral deformity on such images improves the accuracy of pretherapeutically diagnosing the invasion depth of early CRCs [20].

However, when CRC lesions are located on a colorectal fold, it is difficult to estimate quantitatively the depth of lateral deformity based on the tumor geometry on a CT enema image because the lateral deformity on the CT enema image contains not only the lateral deformity caused by both the geometry and invasion of the tumor but also the curved indentation caused by the colorectal fold itself. In clinical practice, though the depth diagnosis of CRC on a colorectal fold using the lateral view of a CT enema or DCBE image is morphologically evaluated by whether the width of colorectal-fold lateral contour is significantly large or not, the evaluation sometimes differs between readers. Accordingly, it is expected that the quantitative evaluation of the width of colorectal-fold lateral contour on CT enema images will improve the accuracy of pretherapeutically diagnosing the invasion depth of CRCs located on a colorectal fold. To our knowledge, there have been no reports on the quantitative evaluation of the width of colorectal-fold lateral contour using a CT enema image in the depth diagnosis of CRCs located on a colorectal fold. In the CT enema image, unlike in the DCBE image, it is possible to evaluate CRCs from any view. Therefore, we devised a lateral split view as a new technique for evaluating the CT enema image and hypothesized that the quantitative evaluation of the width of colorectal-fold lateral contour on CT enema using the lateral split view would be useful for the depth diagnosis of a CRC located on a colorectal fold. Thus, we conducted the present study to investigate the usefulness of determining the invasion depth of CRCs located on a colorectal fold by the quantitative evaluation of the width of colorectal-fold lateral contour on CT enema using lateral split views.

This article has been submitted as a preprint on medRxiv.

## Materials and methods

Subjects

**Figure 1:**
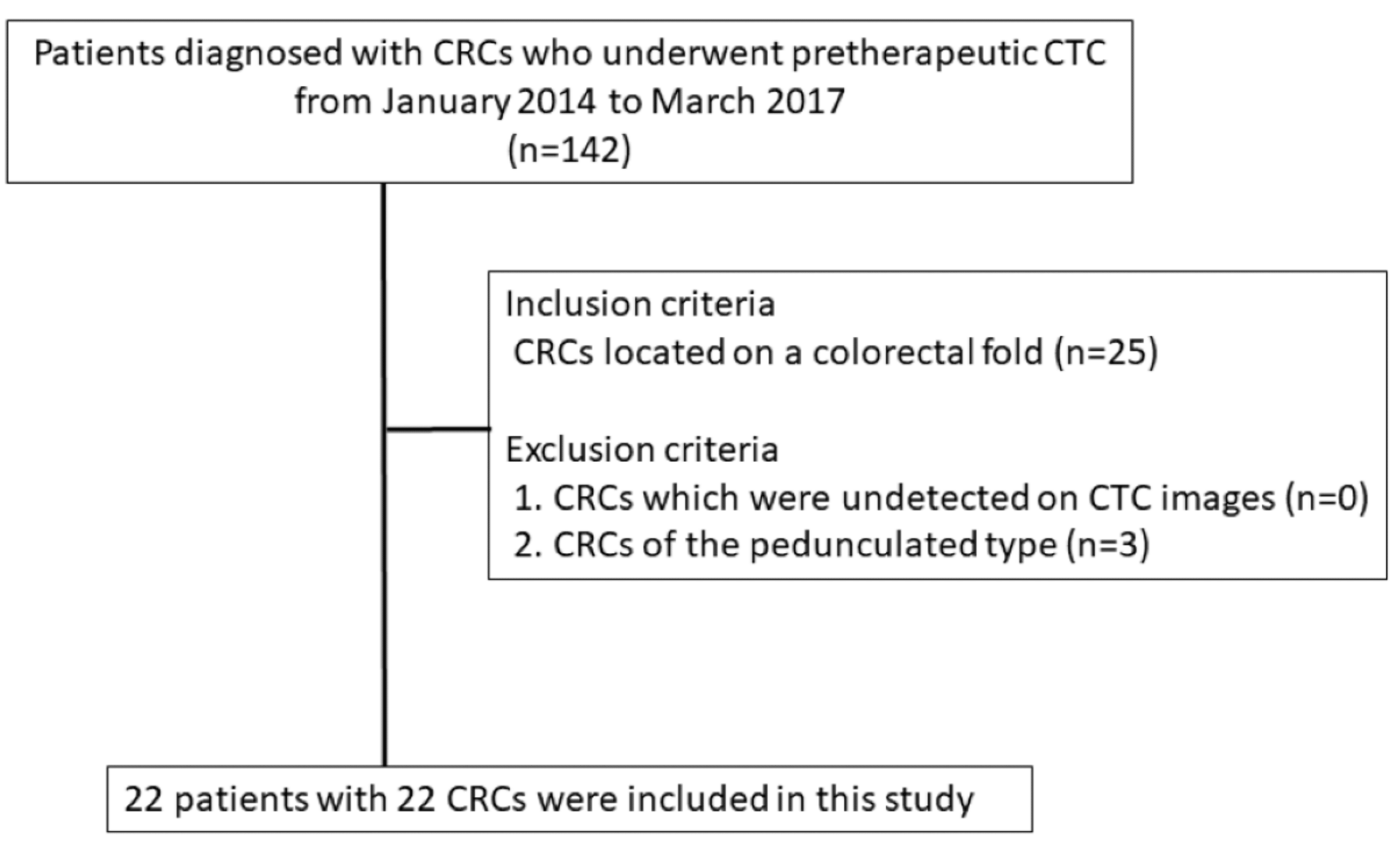
Patient selection flow diagram in this study. CRC: colorectal cancer; CTC: computed tomographic colonography

The initial patient pool consisted of 142 consecutive patients with 150 CRCs who underwent CTC as part of a pretherapeutic examination at Kyushu University Hospital, Fukuoka, Japan, from January 2014 to March 2017. The study included patients with CRCs located on a colorectal fold. Patients with CRC lesions of the pedunculated type were excluded. One of the authors reviewed the CTC and endoscopic images in all subjects. Twenty-five (17.6%) patients with 25 (16.7%) CRCs located on a colorectal fold were enrolled, while three (2.1%) patients with three (2%) CRCs of the pedunculated type located on a colorectal fold were excluded from this study. The final study population included 22 (15.5%) patients (16 (72.7%) males and six (27.3%) females) with 22 (14.7%) CRCs located on a colorectal fold (Figure 1). The mean age of the patients was 65 years (range 47-84 years). Eight (36.4%) CRC lesions were resected endoscopically, and the other 14 (63.6%) CRCs were resected by surgery. This retrospective study was approved by the Kyushu University Medical District Observation Research Ethics Review Committee of our hospital (approval number: 28-345). The requirement for informed consent was waived. The mean interval between the CTC and the endoscopic or surgical resection was 13 days (range 2-27 days). Data was analyzed between January 9, 2014, and March 31, 2017.

Histology

The macroscopic type of CRCs was classified according to the Paris endoscopic classification [21]. T staging was defined according to the Japanese Classification of Colorectal Carcinoma 9th Edition, and early CRCs were classified into three categories: intramucosal carcinoma; T1a-stage, carcinoma invading the superficial submucosa (<1000 μm); and T1b-stage, carcinoma invading the deeper submucosa (≧1000 μm) [5]. In addition, intramucosal cancer diagnosed by Japanese pathologists is diagnosed as high-grade dysplasia by the West pathologists.

CTC procedure

CTC was performed on the same day of and within one hour after a colonoscopy, with bowel preparation using polyethylene glycol. Prior to the scanning, colorectal insufflation with carbon dioxide using a CO2 injector (PROTOCO2L; Bracco, Princeton, NJ, USA) was performed. For all patients, CTC was performed using either a 64-slice multidetector-row computed tomography (MDCT) scanner (Aquilion 64; Canon Medical Systems, Tokyo, Japan) or a 320-slice MDCT scanner (Aquilion One; Canon Medical Systems). The scanning parameters were as follows: 120 kV, 100-300 mA, beam collimation 1 mm, slice thickness 1 mm, reconstruction interval 1 mm, and pitch 0.828 or 0.813. All patients underwent scans in both the supine and prone positions because inadequate distention, residual fluid, or feces in the location of the CRCs make it difficult to evaluate the lesion with the CT enema image. The MDCT data sets were loaded to a workstation (Synapse Vincent, Fujifilm Medical, Tokyo, Japan) where CT enema images and virtual endoscopic images were reconstructed.

Image analysis

**Figure 2:**
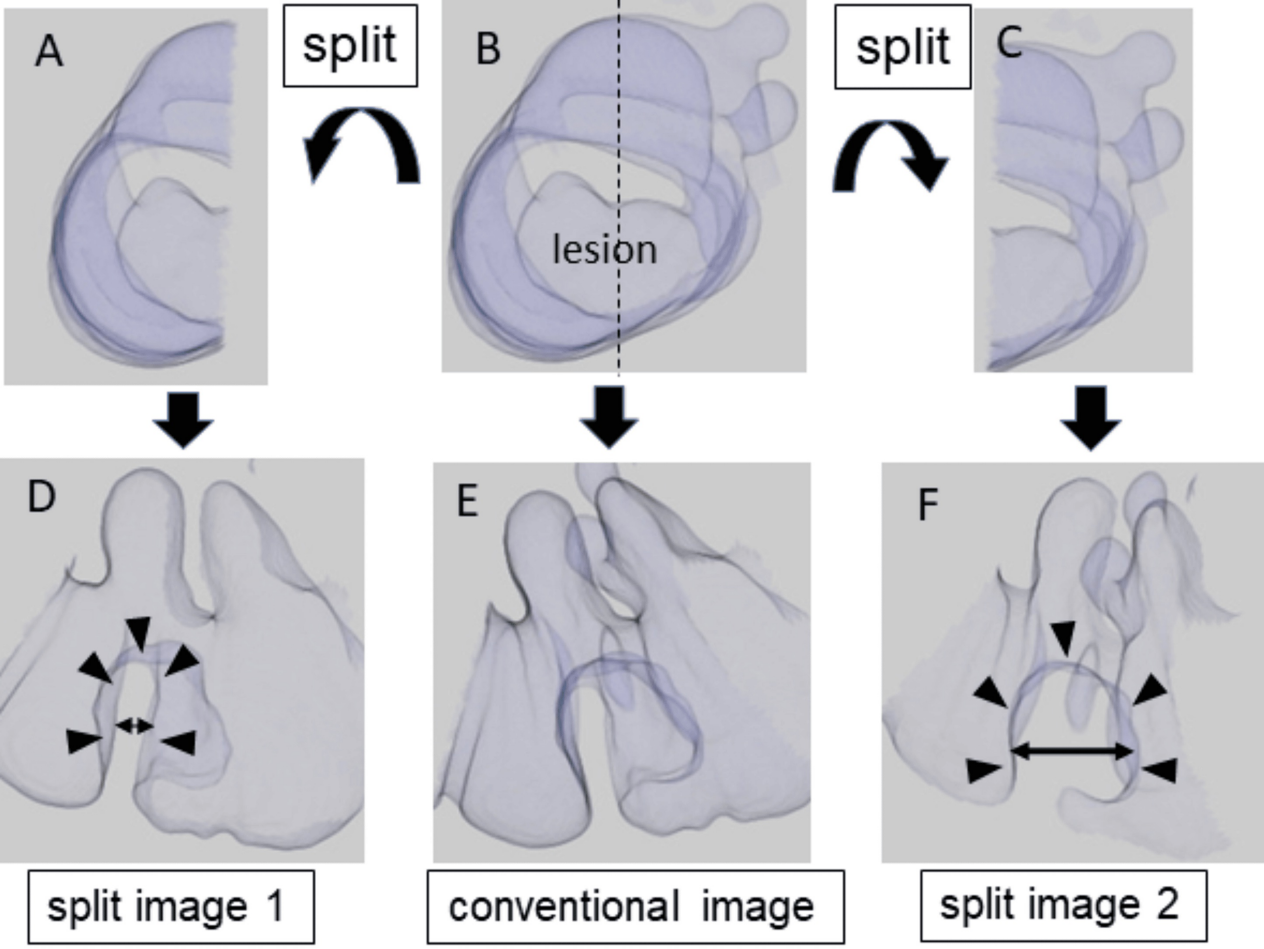
Definition and measurement of the width of colorectal-fold lateral contour using lateral split-view CT enema images of a CRC located on a colorectal fold. First, the split images (A, C) at the central part of the lesion in the cross-sectional view (B) of the CT enema image in the CRCs were extracted. Next, the value of the width (double arrow) of colorectal-fold lateral contour (arrowhead) in the lateral view (D, F) of each split CT enema image was calculated, and the maximum value of the width of colorectal-fold lateral contour was defined as the value of the width of colorectal-fold lateral contour of the CRC located on a colorectal fold. In this case, the value of the width of colorectal-fold lateral contour calculated using split image 2 (F) was defined as the value of the width of colorectal-fold lateral contour of the CRC located on a fold. (E) The lateral view of the conventional CT enema image. This figure was created by the authors for this study. CRC: colorectal cancer; CT enema: computed tomographic air-contrast enema

The definition and measurement methods of the width of colorectal-fold lateral contour on CT enema images are shown in Figure 2. First, we identified CRCs using virtual endoscopic images and CT enema images. Then, we extracted CT enema images in the location of the CRCs using the best distended series that were determined by a qualitative consensus among three gastrointestinal radiologists with 12, 14, and 20 years of experience, respectively. Next, each image was split at the central part of the lesion in the cross-sectional view of the CT enema image, and two images were extracted. Finally, the width of colorectal-fold lateral contour in the lateral view of each split CT enema image was calculated, and the maximum value of the width of colorectal-fold lateral contour was defined as the value of the width of colorectal-fold lateral contour of the CRC located on a colorectal fold. We then compared the maximum width of colorectal-fold lateral contour between the intramucosal/T1a-stage CRCs and the T1b-stage/more deeply invading CRCs. Additionally, we calculated the width of a normal colorectal-fold lateral contour located closest to the CRCs in a lateral view of a CT enema image. These measurements were performed independently by the same three gastrointestinal radiologists who were given no clinicopathological information except for the lesion locations. The mean value of the maximum width of the colorectal-fold lateral contours calculated by the two readers was defined as the most probable width of the colorectal-fold lateral contour.

Statistical analysis

Student's t-test was used to assess the differences in the maximum width of colorectal-fold lateral contour between the intramucosal/T1a-stage and the T1b-stage/more deeply invading CRCs. We also used receiver operating characteristic (ROC) curves to define the optimum cutoff values for the maximum width of colorectal-fold lateral contour to differentiate these two groups of CRCs. Additionally, we calculated the intraclass correlation coefficient (ICC) to evaluate the inter-rater reliability of the assessments on the maximum width of colorectal-fold lateral contour. ICC values of <0.50 were considered to indicate poor reliability, 0.50-0.75 moderate reliability, 0.75-0.90 good reliability, and >0.90 excellent reliability [22]. All statistical analyses were performed using JMP software (JMP version 9.0.2; SAS Institute, Cary, NC, USA). P-values <0.05 were considered significant.

## Results

The clinicopathological characteristics of the CRCs are summarized in Table 1. The average diameter of the CRCs was 18.7±2.0 mm (range 8-38 mm). Six (27.3%) lesions were intramucosal CRCs, six (27.3%) were T1-stage CRCs, four (18.2%) were T2-stage CRCs, and six (27.3%) were T3/T4-stage CRCs. There were three (13.6%) lesions of type 0-Is, six (27.3%) lesions of type 0-Is+IIc, one (4.5%) lesion of type 0-IIa, four (18.2%) lesions of type 0-IIa+IIc, three (13.6%) lesions of type 1, and five (22.7%) lesions of type 2. There was no significant difference in age, sex, tumor size, tumor location, or tumor differentiation between the intramucosal/T1a-stage CRC group and the T1b-stage/more deeply invading CRC group.

**Table 1:** Characteristics of the 22 patients with 22 CRCs. CRC: colorectal cancer

Clinicopathological factors		Intramucosal/T1a-stage CRCs (n=8)	T1b-stage/more deeply invasive CRCs (n=14)	P-value
Age (years) (range)		73.1 (65-81)	66.3 (44-81)	0.1054
Sex	Men	6 (75%)	10 (71.4%)	0.8647
	Women	2 (25%)	4 (28.6%)	
Tumor location	Right-sided colon	6 (75%)	8 (57.1%)	0.4266
	Left-sided colon and rectum	2 (25%)	6 (42.9%)	
Tumor diameter (mm) (mean±SD）		19.4 (8-38)	18.8 (11-33)	0.6982
Tumor differentiation	Differentiated type	8 (100%)	11 (78.6%)	0.1744
	Undifferentiated type	0 (0%)	3 (21.4%)	

**Figure 3:**
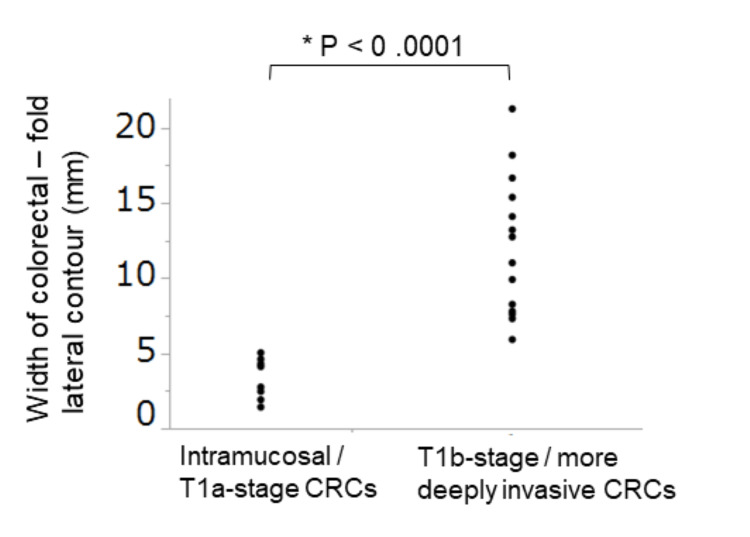
Comparison of the maximum values of the width of colorectal-fold lateral contour between the intramucosal/T1a-stage CRC group and the T1b-stage/more deeply invading CRC group. There was a significant difference in the maximum values of the width of colorectal-fold lateral contour between the intramucosal/T1a-stage CRCs and T1b-stage/more deeply invading CRCs (3.3±1.4 mm and 12.1±4.6 mm, respectively; p<0.0001). CRC: colorectal cancer

**Figure 4:**
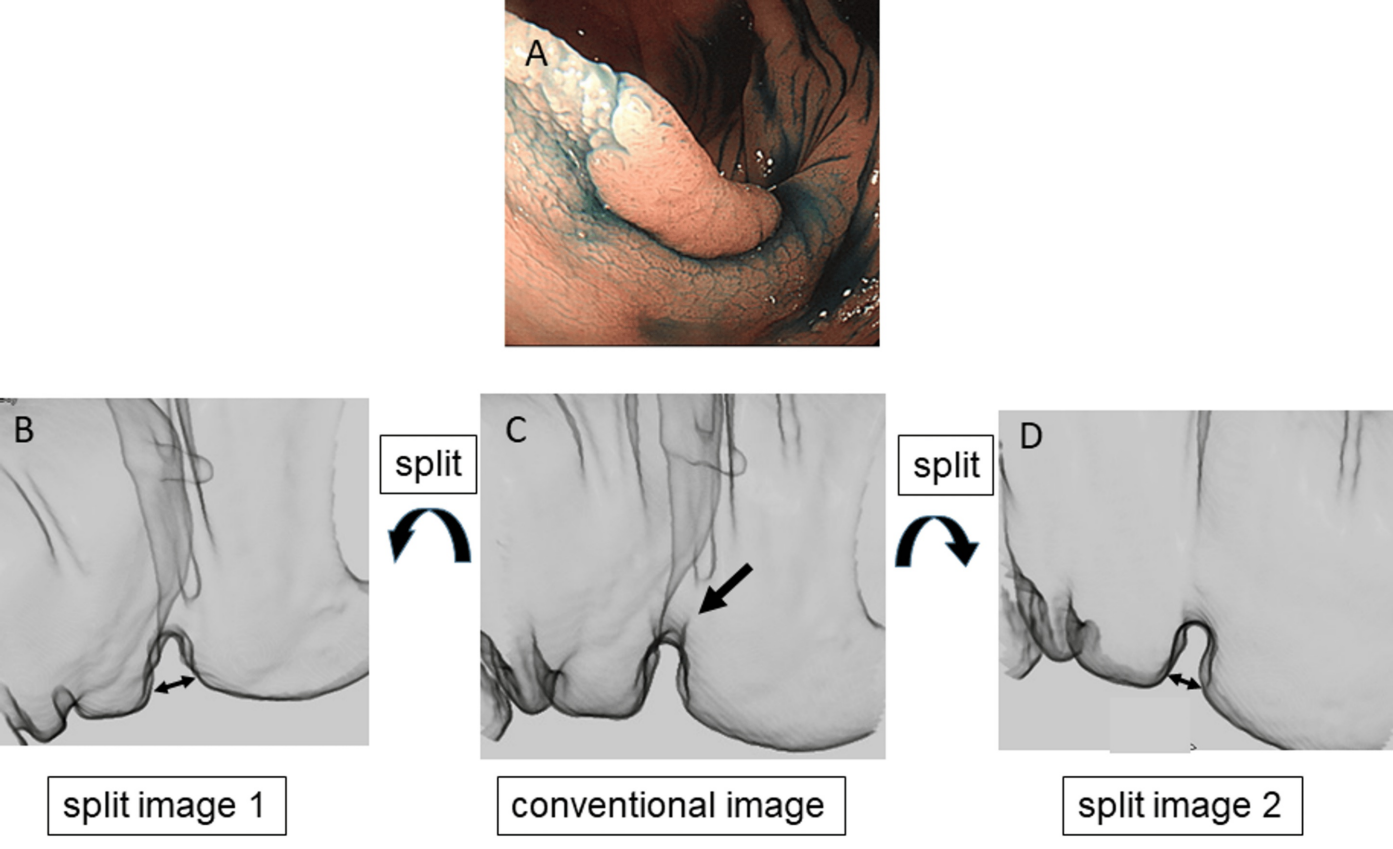
A 69-year-old man with intramucosal ascending colon cancer, type 0-IIa. (A) Optical endoscopic image shows an elevated lesion located on a colorectal fold. (C) The lateral view of the conventional CT enema image. The values of the width (double arrow) of colorectal-fold lateral contour on the lateral split-view CT enema images 1 (B) and 2 (D) in the CRC located on a fold (arrow) were 2.5 mm and 2.4 mm, respectively. Accordingly, the maximum value of the width of colorectal-fold lateral contour was diagnosed as 2.5 mm. This figure was created by the authors for this study. CRC: colorectal cancer; CT enema: computed tomographic air-contrast enema

**Figure 5:**
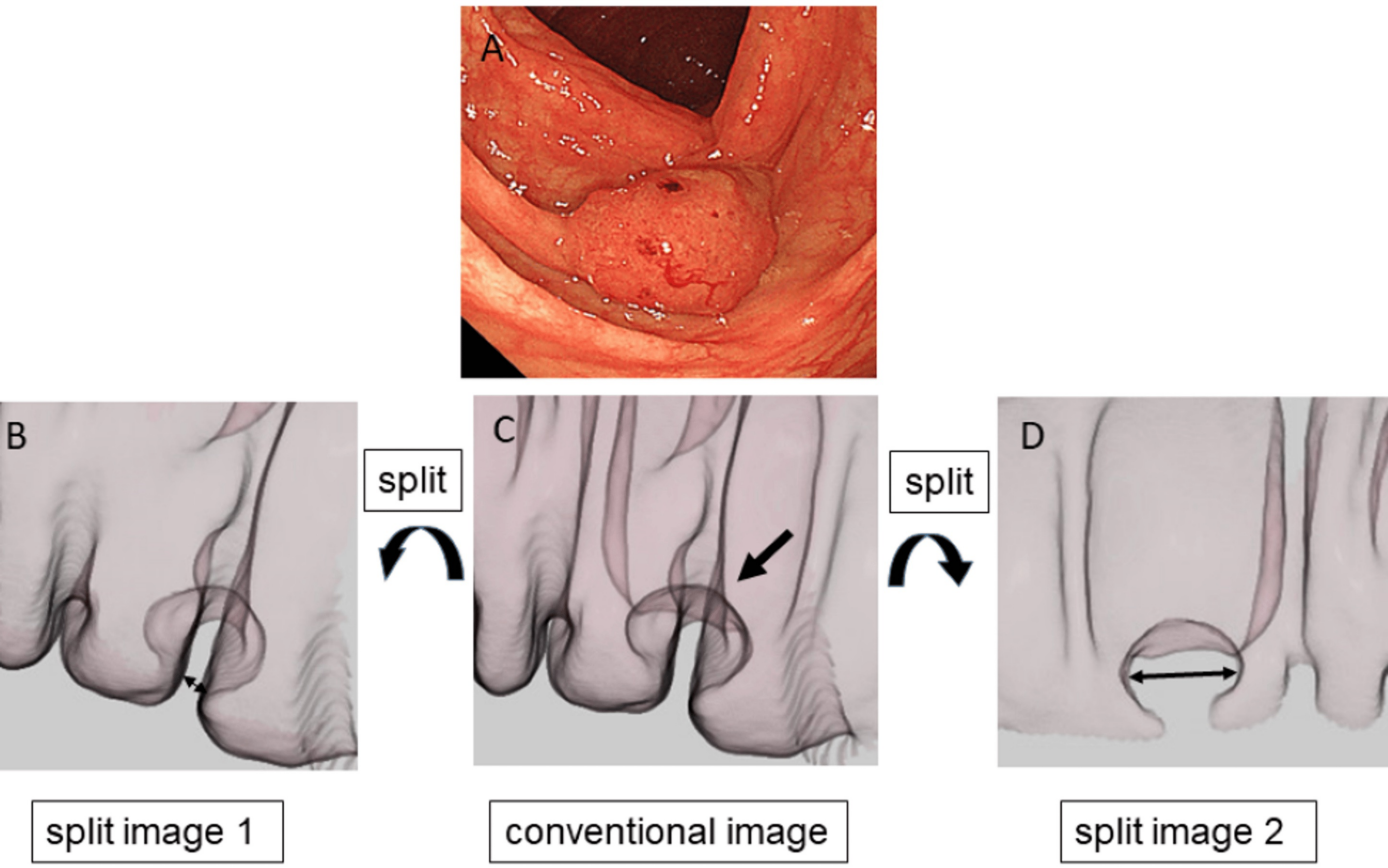
A 57-year-old woman with T1b-stage ascending colon cancer, type 0-Is. (A) Optical endoscopic image shows an elevated lesion located on a colorectal fold. (C) The lateral view of the conventional CT enema image. The values of a width (double arrow) of colorectal-fold lateral contour on the lateral split-view CT enema images 1 (B) and 2 (D) in the CRC located on a colorectal fold (arrow) were 2 mm and 11 mm, respectively. Accordingly, the maximum value of the width of colorectal-fold lateral contour was diagnosed as 11 mm. This figure was created by the authors for this study. CRC: colorectal cancer; CT enema: computed tomographic air-contrast enema

There was a significant difference in the maximum width of colorectal-fold lateral contour between the intramucosal/T1a-stage and T1b-stage/more deeply invading CRCs (3.3±1.4 mm and 12.1±4.6 mm, respectively; p<0.05) (Figure 3). Figure 4 and Figure 5 show representative intramucosal CRC and T1b-stage CRC cases, respectively. The ROC analysis showed that the optimum cutoff value of the maximum width of colorectal-fold lateral contour for differentiating intramucosal/T1a-stage CRCs from T1b-stage/more deeply invading CRCs was 6 mm, with an area under the curve (AUC) of 0.991. If CRCs with the maximum width of colorectal-fold lateral contour of >6 mm were interpreted as T1b-stage/more deeply invading CRCs, the sensitivity and specificity for differentiating intramucosal/T1a-stage CRCs from T1b-stage/more deeply invading CRCs were 92.9% and 87.5%, respectively. The ICC value for the measurement of colorectal-fold lateral contour was 0.949, which met the definition for excellent reliability. The mean width of a normal colorectal-fold lateral contour was 2.3±0.6 mm.

## Discussion

In this study, we reported for the first time the usefulness of quantitatively evaluating the width of colorectal-fold lateral contour on CT enema images to diagnose the invasion depth of CRCs located on a colorectal fold. A lateral deformity on a CT enema or DCBE image is one of the most important indicators for the diagnosis of invasive CRC, in addition to deep depression, protrusion in depression, and fold convergency [10,13,14,23]. In the morphologic evaluation of lateral deformity on CT enema or DCBE images in CRCs located on a colorectal fold, CRCs with "a normal width of colorectal-fold lateral contour" are diagnosed as intramucosal/T1a-stage CRCs, while those with "a large width of colorectal-fold lateral contour" are diagnosed as T1b-stage/more deeply invading CRCs. A large width of colorectal-fold lateral contour is thought to result primarily from poor intestinal distension due to tumor infiltration including associated desmoplastic reaction. However, in our experience, readers morphologically evaluating the widths of colorectal-fold lateral contour on CT enema or DCBE images sometimes have differing opinions as to whether or not a width of colorectal-fold lateral contour is significantly large. One of the reasons for the disagreement is that lateral views of CT enema or DCBE images in CRCs located on a colorectal fold are composed of complex lines,  i.e., the indentation of the colorectal fold, tumor surface, and tumor base. CT enema imaging is better than DCBE imaging for obtaining a precise and secure lateral view [14,23]. Furthermore, in the present study, it was possible to simplify these complex lines using lateral split-view CT enema images and to calculate objectively the width of colorectal-fold lateral contour.

On the other hand, previous studies have reported that lateral deformities based on tumor geometry were detectable in colorectal polyps on DCBE and CT enema images [11,12,18]. In CRCs, the depth of the lateral deformity based on tumor geometry depends on the size of the tumor based on a CS-MPR image. Namely, the larger the size of the tumor base is, the larger the value of the depth of lateral deformity based on the tumor geometry will be. It is also necessary to take account of the lateral deformity based on tumor geometry in the depth diagnosis due to lateral deformities in CRCs located on a colorectal fold. However, it is difficult to estimate quantitatively the depth of lateral deformities based on the tumor geometry on CT enema images in CRCs located on a colorectal fold. Nevertheless, in the present study, the diagnostic accuracy of invasion depth for CRCs located on a colorectal fold using the width of colorectal-fold lateral contour on a CT enema image was high, even though we could not estimate quantitatively the depth of lateral deformity based on tumor geometry. In the depth diagnosis of CRCs located on a colorectal fold using the quantitative evaluation of lateral deformity on a CT enema image, it may not be very important to estimate quantitatively the depth of lateral deformity based on tumor geometry. Endoscopic ultrasound is also useful for the determination of the invasion depth for CRCs, with a diagnostic accuracy of approximately 90%, but CRCs located on a colorectal fold are often difficult to image on endoscopic ultrasound [24]. Therefore, the integration of other imaging modalities such as endoscopic ultrasound or conventional CTC would not further improve diagnostic performance in the invasion depth of CRCs located on a colorectal fold. The diagnosis of invasion depth for CRCs using the lateral split view of a CT enema image, in particular, could play an important diagnostic role for CRCs located on a colorectal fold. The results of the present study show that the measurement of the width of colorectal-fold lateral contour using a lateral split-view CT enema image can be useful to differentiate intramucosal/T1a-stage CRCs from T1b-stage/more deeply invading CRCs with high diagnostic accuracy. Namely, it was suggested that CRCs with a maximum width of colorectal-fold lateral contour of 6 mm or less should be diagnosed as intramucosal/T1a-stage CRCs and endoscopic treatment should be considered, whereas those with a maximum width of more than 6 mm should be diagnosed as T1b-stage/more deeply invading CRCs and surgery should be considered.

This study had some limitations. First, there are discrepancies in the pathological diagnosis of intramucosal colorectal neoplasia between the West and Japan. However, this discrepancy does not matter much when deciding on a treatment strategy. Second, selection bias is a possible limitation because one of the authors reviewed the CTC and endoscopic images in all subjects. Third, the present study was evaluated by only three experienced gastrointestinal radiologists. It is necessary to verify whether the method of measuring the width of the lateral contour of the colorectal fold is reproducible by non-expert radiologists as well. Fourth, the small number of subjects and the fact that this was a single-center retrospective study are also limitations. Further, a large-scale multicenter prospective study is also needed to confirm our initial findings and determine the diagnostic accuracy for the width of colorectal-fold lateral contour.

## Conclusions

In the depth diagnosis of CRC located on a colorectal fold, the use of a lateral split-view CT enema image can provide higher diagnostic accuracy for the quantitative evaluation of the width of colorectal-fold lateral contour. This may be a new diagnostic approach for the determination of the invasion depth of CRCs located on a colorectal fold.
